# A seasonal periodicity in relapses of multiple sclerosis? A single-center, population-based, preliminary study conducted in Bologna, Italy

**DOI:** 10.1186/1471-2377-10-105

**Published:** 2010-11-01

**Authors:** Fabrizio Salvi, Ilaria Bartolomei, Michael H Smolensky, Angelo Lorusso, Elena Barbarossa, Anna Maria Malagoni, Paolo Zamboni, Roberto Manfredini

**Affiliations:** 1 Department of Neuroscience, Multiple Sclerosis Center, Bellaria Hospital, Bologna, Italy; 2 Department of Biomedical Engineering, the University of Texas at Austin, USA; 3 Vascular Diseases Center, University of Ferrara, Italy; 4 Department of Internal Medicine, Hospital of the Delta, Azienda Unità Sanitaria Locale, Ferrara, Italy; 5 Department of Clinical and Experimental Medicine, Clinica Medica and Vascular Diseases Center, University of Ferrara, Italy

## Abstract

**Background:**

Temporal, i.e., 24-hour, weekly, and seasonal patterns in the occurrence of acute cardiovascular and cerebrovascular events are well documented; however, little is known about temporal, especially seasonal, variation in multiple sclerosis (MS) and its relapses. This study investigated, by means of a validated chronobiological method, whether severe relapses of MS, ones requiring medical specialty consultation, display seasonal differences, and whether they are linked with seasonal differences in local meteorological variables.

**Results:**

We considered 96 consecutive patients with severe MS relapse (29 men, 67 women, mean age 38.5 ± 8.8 years), referred to the Multiple Sclerosis Center, Bellaria Hospital, Bologna, Italy, between January 1, 2007 and December 31, 2008. Overall, we analyzed 164 relapses (56 in men, 108 in women; 115 in patients aged < 40 years, 49 in patients ≥40 years). Relapses were more frequent in May and June (12.2% each) and the least frequent in September (3.7%). Chronobiological analysis showed a biphasic pattern (major peak in May-June, secondary peak in November-December, *p *= 0.030). Analysis of monthly mean meteorological data showed a significant seasonal pattern in ambient temperature (peak in July, *p *< 0.001), relative humidity (peak in January, *p *< 0.001), and wind speed (peak in June, *p *= 0.011).

**Conclusions:**

In this Italian setting, we found a biphasic pattern (peaks in spring and autumn) in severe MS relapses requiring medical consultation by doctors of the MS specialty center, apparently unrelated to meteorological variables. Confirmations of the findings on larger multi-center populations residing in different climatic conditions are needed to further explore the potential seasonality of MS relapses and associated environmental triggers.

## Background

Several medical conditions, in particular acute cardiovascular and cerebrovascular ones, exhibit prominent temporal patterns, i.e., 24-hour, weekly, and seasonal patterns in exacerbation and mortality. For example, a seasonal pattern, characterized by an autumn-winter excess of onset, has been recently confirmed in the Emilia-Romagna region of Italy for acute myocardial infarction [1], aortic diseases [2], and transient ischemich attack [3]. Little is known, however, about the seasonality of many other types of diseases, especially neurological ones, including multiple sclerosis (MS) and its relapses, particularly in Italy. Thus, we investigated, by means of a validated chronobiological statistical method, whether severe relapses of MS display seasonal differences, and whether they are associated with temporal patterns in local meteorological variables.

## Methods

### Data collection

We evaluated all consecutive cases of relapse in MS patients referred to the Multiple Sclerosis Center, Bellaria Hospital, Bologna, Italy, between January 1, 2007 and December 31, 2008. The study was approved by the local ethics committee. The definition of relapse is the occurrence of a new neurological deficit of at least one-day's duration that is unrelated to any medical condition, such as fever, that might be causal of the exacerbated MS symptoms. We selected this definition since for clinical purposes it is generally agreed that an attack (or relapse), whether defined by subjective report or by objective observation, should last at least 24 hours [4]. This definition also ensures that the event is not a pseudoattack that might be due to change in core body temperature with infection. Whereas reports of attacks may be provided by subjective retrospective patient narratives, objective clinical findings of a lesion are required to make the diagnosis of MS. Single paroxysmal episodes (eg, a tonic spasm) do not constitute a relapse, but multiple episodes occurring over not less than 24 hours do [4].

In our MS Center, every patient is eligible to undergo a neurological evaluation within two days of a suspected relapse, and an advertised mobile phone number is dedicated to request appointments for such. Each relapse event was checked by at least one of the neurology investigators to ensure satisfaction of inclusion criteria. Frequent attacks when experienced by the same patient were considered separate relapses *only *when the interval between each exceeded one month. Patients were also categorized into two subgroups according to age (< 40 or ≥40 years). This age criterion was chosen since relapses in MS seem to be age dependent, with most occurring in persons between 20 and 40 years of age [5]. Moreover, being a male and experiencing late-age onset of MS are related to poor prognosis [6].

Each relapse was categorized into one of twelve 1-month intervals according to the day and month of the onset of symptoms, based on reports systematically obtained from patients, relatives or other care-takers. In addition, we collected data on the average monthly ambient temperature (°C), relative humidity (%), wind speed (m/sec), and rain precipitation (mm) using instrumentation manufactured by Vaisala Oyi (Helsinki, Finland) and maintained by the Agenzia Regionale Prevenzione e Ambiente (ARPA) of the Emilia-Romagna region, Bologna, Italy (active since May 2004), located at longitude 11.32872, latitude 44.500752, and altitude 48 m above sea level. Meteorological measures were collected daily at 13:00 h and averaged to derive the monthly means of each variable.

### Statistical analysis

We performed the main statistical analysis by applying partial Fourier series with up to four harmonics (Chronolab, free download at: http://www.tsc.uvigo.es/BIO/) [7] to the time series of total MS cases, subgroups categorized by sex and age (< 40 and ≥40 years), and each meteorological variable. This method of analysis selects the harmonic (or the combination of harmonics) that best explain the variance of the time series data. The percentage of the overall variability of the data about the arithmetic mean attributable to the approximated cosine curve of a given period by the method of least squares) estimates the goodness of fit of the model, and the F-test statistic serves to test the zero-amplitude null hypothesis (absence of periodicity). The parameters calculated for the overall one-year in period (i.e., 8.766-hour) cosine approximation of the time series are: the "percentage of rhythm" (PR) - percentage of overall variability of data about the arithmetic mean attributable to the fitted rhytmic function; the midline estimated statistic of rhythm (MESOR) - here the rhythm-adjusted annual mean; amplitude - one half the difference between the absolute maximum and minimum of the fitted curve; and the peak time (acrophase) of the occurrence of the absolute maximum value during the year expressed in degrees (360° = 365 years or 1 day = 0.9863°) as a negative value from the zero-time reference, midnight December 31. Significance levels were assumed for *p *< 0.05.

## Results

**Figure 1:**
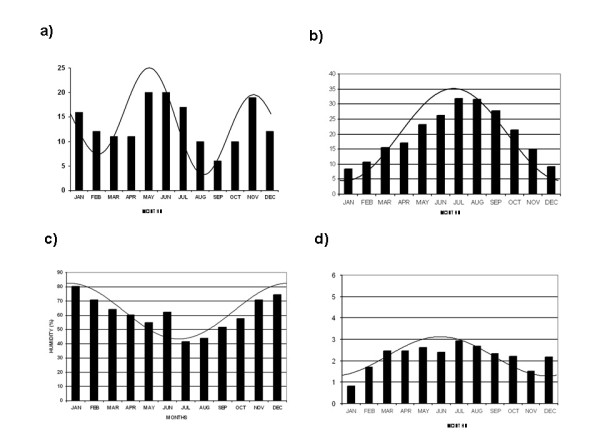
**Frequency of relapses of multiple sclerosis by month of the year (a) and average monthly data of ambient temperature in °C (b), relative humidity as % (c), and wind speed as m/sec (d)**.

Overall, we considered 96 patients (29 men, 67 women, mean age 38.5 ± 8.8 years), who experienced in total 164 relapses (56 in men, 108 in women; 115 in persons aged < 40 years, 49 in persons aged ≥40 years). In the absence of any specific temporal patterning of relapses, 8.33% of the total number of cases would be expected to occur each month of the year. As shown in Figure 1-a, the distribution by month of the relapses for the entire group was highest in May and June (12.2% each) and the lowest in September (3.7%). Time series analysis (Table 1) yielded a cyclic biphasic pattern, characterized by a major peak in MS in May and a secondary peak in November (*p *= 0.03). Older patients (≥40 years of age) exhibited the same temporal pattern (*p *= 0.025), whereas younger patients showed only a non-statistically significant singled peak (may) seasonal trend (*p *= 0.065). No significant periodicity was found in subgroups categorized by sex. The time series analysis of meteorological variables (Table 1) revealed significant seasonal variation with respective peaks in July for ambient temperature (Figure 1-1b, *p *< 0.001), in January for relative humidity (Figure 1-1c, *p *< 0.001), and in June for wind speed (Figure 1-1d, *p *= 0.011). Rain precipitation did not exhibit any significant seasonal variation.

**Table 1:** Chronobiologic analysis of seasonal distribution of multiple sclerosis relapses, and meteorological data

	N	Period (hours)	PR (%)	MESOR ± SE	Amplitude	Acrophase	p
Total relapses	164	8766.00	10.7				N.S.
		4383.00	64.0				0.012
		Overall	74.7	13.69 ± 0.83	6.42	-142° *(May)*	0.030
							
Females	108	8766.00	12.1				N.S.
		4383.00	29.6				N.S.
		Overall	41.7	9.01 ± 0.95	3.62	-139°	N.S.
							
Males	56	8766.00	2.3				N.S.
		4383.00	35.1				N.S.
		Overall	37.4	4.68 ± 0.87	2.81	-144°	N.S.
							
< 40 yrs	115	8766.00	2.7				N.S.
		4383.00	64.9				0.021
		Overall	67.6	9.60 ± 0.72	4.37	-142° *(May)*	0.065
							
≥40 yrs	49	8766.00	41.0				0.031
		4383.00	34.9				0.043
		Overall	75.8	4.09 ± 0.26	2.11	-141° *(May)*	0.025
							
Temperature (°C)		8766.00	97.4	15.4 ± 0.39	10.05	-188.8 ± 3. 2 *(July)*	< 0.001
Humidity (%)		8766.00	81.2	61.0 ± 1.65	14.57	-3.3 ± 9.2 *(January)*	< 0.001
Wind speed (m/sec)		8766.00	63.1	2.20 ± 0.11	0.63	-164.7 ± 14.6 *(June)*	0.011
Rain precipitation (mm)		8766.00	0.7	1.81 ± 0.47	0.17	-20.8 ± 227.1	N.S.

Abbreviations (see text for details) derived by Cosinor analysis of the time series data: **PR **: percentage of rhythm - **MESOR**: midline estimated statistic of rhythm (the rhythm-adjusted mean over the time period analyzed, i.e., the monthly mean for a cycle of one year) - **Amplitude**: one-half the difference between the absolute maximum and minimum of the fitted curve - **Acrophase**: the absolute time of the peak expressed in degrees, with 360° = 12 months or 1 day = 0.9863, as a negative value (i.e., a delay) in reference to midnight December 31. P value derived by the F-test statistic of the null hypothesis that the amplitude of the fitted cosine approximation of a given period = 0^7^

## Discussion

In this Italian setting (Bologna, Emilia-Romagna region), we found a seasonal pattern for MS relapses, apparently unrelated to meteorological variables, characterized by biphasic spring-autumn peaks (main peak in May, secondary peak in November) and summer trough, especially in older (≥40 years) patients, and a trend for a single peak in May in younger subjects.

Seasons seem to play a role in MS. For example, an association between month of birth and the risk of later developing of MS have been documented in studies conducted in Canada, Great Britain, Denmark and Sweden [8,9], with persons born in May [8] and June [9] exhibiting significantly increased risk of MS.

Multiple sclerosis is an inflammatory demyelinating disease of the central nervous system of unknown pathogenesis, although environmental [10], genetic [11], infectious [12], toxic [13], nutritional [14], hormonal [15], and venous vascular [16] factors have been studied as plausible risk factors. Moreover, exposure to ionizing radiation [17], and organic solvents [18], including volatile anaesthetic agents [19], may constitute additional risk factors. Thus, the disparate expression of the disease itself, characterized either by a chronic progressive or a relapsing/remitting clinical picture, perhaps helps to explain differences between the available data on its seasonal variation. Environmental factors that can potentially play a role in risk of MS development or progression include viral infections (cytokines?) and vitamin D levels (sun exposure?), both of which exhibit seasonal variation [20,21].

Ogawa et al. [22] observed that relapses of MS patients were significantly more frequent in the warmest and coldest months of the year. Stewart et al. [23] observed that untreated MS (relapsing/remitting) cases showed a summer excess of interleukin-10, and Balashov et al. [24] reported a significantly increased interferon production in the autumn and winter compared to the spring and summer in chronic progressive MS, with maximum values of T-cell activation (assessed in terms of tumor necrosis factor and interferon levels) found during autumn by Killestein et al. [25]. A population-based study conducted in Southern Tasmania [26] detected an inverse association between MS relapse rate and erythemal ultraviolet radiation (EUV) and vitamin D [25(OH)D] levels, suggesting a role of EUV exposure.

Very recently, contrasting evidence on the seasonality of relapsing MS has been reported as well. A large Portuguese retrospective study of relapsing/remitting patients (414 relapses occurring in 249 consecutive patients studied between January 1, 2004 and December 31, 2007) [27 found no significant differences between months or seasons, and no correlation between relapse frequency and weather factors, including maximum and minimum temperature, humidity, and atmospheric pressure. The results of a 3-year Israeli study (2001-2003) of 235 patients [28] also found no significant correlation between number of relapses and season or month, and no significant impact of meteorological parameters on relapses. On the other hand, a strong seasonal pattern in subclinical MS activity was found in the United States by Meier et al. [29], who investigated the seasonal prevalence of MS disease activity in terms of appearance of new lesions found in serial T2-weighted MRI (n = 939 separate MRI examinations) of a cohort of 44 untreated patients. They also tested for associations of seasonality with recorded meteorologic data, i.e., ambient temperature, solar radiation and precipitation, in the Boston vicinity between1991-1993 when the MRI examinations were made. The likelihood of new T2 activity was 2-3 times higher between the months of March-August than during the other months of the year, with a strong correlation with solar radiation and with disease intensity being elevated during the summer season.

## Conclusions

Even with its evident limitations, i.e., single-center setting, limited population size, and relatively small number of events, to the best of our knowledge, this is the first study to utilize methods specific to the field of medical chronobiology to explore the seasonality of MS pathophysiology. The possible relationships between seasons and environmental factors deserve further exploration on larger multi-center populations residing in different climates. Furthermore, the role played by environmental factors and endogenous human biological rhythms requires more-in-depth investigation, since it is plausible that seasonal variation of immune status [30] and responsiveness might play a role as well. It is suggestive, in fact, that MS and allergic respiratory diseases exhibit an inverse relationship, with MS tending to be less severe when associated with allergic respiratory diseases [31]. Finally, recent findings on seasonal variation in MS lesion activity as determined by MRI examination indicate that future clinical trials should include such assessments in their design along with serial measurements of those environmental factors associated in previously conducted studies with seasonal patterns in MS [32].

## Competing interests

The authors declare that they have no competing interests.

## Authors' contributions

FS, IB, AL, PZ, RM: conceived of the study, participated in the design and coordination of the study, performed the statistical analysis, and participated to draft and critically revised the manuscript. MHS, EB, MAM: participated in the design of the study, obtained data, participated in the statistical analysis, and participated to draft the manuscript. All authors read and approved the final manuscript.

## Pre-publication history

The pre-publication history for this paper can be accessed here:

http://www.biomedcentral.com/1471-2377/10/105/prepub
